# PLASMA PROTEOMIC SIGNATURE OF DEVELOPMENT AND PROGNOSIS OF SARCOPENIA

**DOI:** 10.1093/geroni/igae098.1720

**Published:** 2024-12-31

**Authors:** Chenkai Wu, Jinhui Liao, Jianhong Xu

**Affiliations:** Duke Kunshan University, Kunshan, Jiangsu, China (People’s Republic); Duke Kunshan University, Kunshan, Jiangsu, China (People’s Republic); Duke Kunshan University, Kunshan, Jiangsu, China (People’s Republic)

## Abstract

Sarcopenia, characterized by muscle loss and functional decline, poses a significant health concern in aging populations. Understanding the proteomic signatures associated with the development and prognosis of sarcopenia is crucial for targeted management. Data from 52,689 participants (aged 39-70 years) in the UK Biobank were analyzed, with 2,920 proteins assayed using Olink. We used the multinomial logistic regression to identify proteins cross-sectionally associated with sarcopenia at baseline; Cox regression was used to examine proteins associated with all-cause mortality among 2,886 individuals who were sarcopenic at baseline. Subsequently, we used the LASSO regression to screen for important proteins for predicting death among sarcopenic persons and the light gradient boosting machine model to determine relative importance of these proteins. Pathway analysis was performed using Enrichr. A total of 738 proteins were cross-sectionally associated with sarcopenia at baseline, while 627 proteins were significantly associated with mortality among initially sarcopenic individuals (Bonferroni-corrected p-value < 0.05). LASSO regression identified 306 and 65 proteins associated with sarcopenia status and death, respectively. Among the 34 overlapping proteins, GDF15, WFDC2, EDA2R, and CHRDL1 were the top signals. The prediction model with these 34 proteins had a C-statistic=0.81 and performed better than a base model with key socio-demographic variables (net reclassification index=0.27). Differential expression analysis revealed enrichment of proteins in cellular signaling, extracellular matrix remodeling, cancer initiation and progression, and metabolic regulation pathways. Identification of proteomic signatures of sarcopenia provides insights into its pathogenesis and prognosis. The refined predictive models offer potential clinical utility in personalized management strategies for sarcopenia.

